# Development of EST‐SSR markers in *Saxifraga sinomontana* (Saxifragaceae) and cross‐amplification in three related species

**DOI:** 10.1002/aps3.11269

**Published:** 2019-06-12

**Authors:** Yan Li, Liu‐Kun Jia, Fa‐Qi Zhang, Zhi‐Hua Wang, Shi‐Long Chen, Qing‐Bo Gao

**Affiliations:** ^1^ Key Laboratory of Adaptation and Evolution of Plateau Biota Northwest Institute of Plateau Biology Chinese Academy of Sciences Xining 810008 People's Republic of China; ^2^ University of Chinese Academy of Sciences Beijing 100039 People's Republic of China; ^3^ Qinghai Provincial Key Laboratory of Crop Molecular Breeding Xining 810008 People's Republic of China

**Keywords:** EST‐SSR markers, *Saxifraga congestiflora*, *Saxifraga heleonastes*, *Saxifraga sinomontana*, *Saxifraga tangutica*, transcriptome

## Abstract

**Premise:**

*Saxifraga sinomontana* (Saxifragaceae) is a widespread alpine species in the Qinghai–Tibetan Plateau and its flanking mountains. We developed a set of expressed sequence tag–simple sequence repeat (EST‐SSR) markers to investigate the genetic diversity and evolutionary history of the species.

**Methods and Results:**

We initially designed 50 EST‐SSR markers based on transcriptome data of *S. sinomontana*. Nineteen of 50 loci (38%) were successfully amplified, 13 of which were polymorphic. These were tested on 71 individuals from four populations. Three to 18 alleles per locus were detected, and the levels of observed and expected heterozygosity ranged from 0.2817 to 0.9155 and 0.2585 to 0.8495, respectively. In addition, cross‐amplification was successful for all 13 loci in three congeneric species, *S. tangutica*,* S. heleonastes*, and *S. congestiflora*.

**Conclusions:**

These EST‐SSR markers will be useful for studying the genetic diversity of *S. sinomontana* and disentangling the phylogenetic relationships of related species.

The genus *Saxifraga* L. (Saxifragaceae) consists of approximately 500 species that are mainly distributed in the mountainous regions of Europe and Asia (Pan et al., 2001; Gao et al., 2015; Tkach et al., 2015), including the Qinghai–Tibetan Plateau (QTP) and Hengduan Mountains region (HDM), which is a biodiversity hotspot of *Saxifraga* (Pan et al., 2001). *Saxifraga sinomontana* J. T. Pan & Gornall is a widespread perennial herb in the QTP and its peripheral regions. It prefers scrub, alpine/marshy meadows, or calcareous crevices at elevations of 2700–5300 m (Pan et al., 2001). Diagnostic features of the species are pedicels with sparsely brown crisped villi and erect sepals that are covered by crisped villi marginally and abaxially (Pan et al., 2001). However, *S. sinomontana* is an extraordinarily variable species in morphology, as described in the *Flora of China* (Pan et al., 2001), as well as according to our long‐term field surveys, which have demonstrated that gradations in traits are common in the species (e.g., plant height, number of flowers). *Saxifraga sinomontana* has been the focus of recent systematics research (Gao et al., 2015; Tkach et al., 2015), and Li et al. (2018) revealed that this species possesses a high level of genetic diversity, which may be the result of Quaternary climatic oscillations.

Microsatellite markers for *S. sinomontana* and its closely related species are not available at present, which limits the development of genetic studies. Due to the advantages of codominance, high polymorphism, and widespread distribution throughout the genome (Bouck and Vision, 2007), expressed sequence tag–simple sequence repeat (EST‐SSR) markers are widely applied in genetic diversity research. Moreover, EST‐SSR markers are relatively easy and inexpensive to develop, and more transferable among closely related species than genomic SSRs (Bouck and Vision, 2007; Ellis and Burke, 2007). In this study, we developed 13 EST‐SSR markers for further population genetic studies of *S. sinomontana*. Additionally, we evaluated the transferability of these markers in the three sympatric and congeneric species *S. tangutica* Engl., *S. heleonastes* Harry Sm., and *S. congestiflora* Engl. & Irmsch.

## METHODS AND RESULTS

Fresh leaf tissue of *S. sinomontana* was collected from Yushu, Qinghai Province, China (Appendix 1), and was frozen in liquid nitrogen before storage at −80°C. Total RNA was extracted using the protocol described by Kumar and Singh (2012). The mRNA was then purified from total RNA using poly‐T oligo‐attached magnetic beads and fragmented into short fragments. cDNA libraries were prepared for 150–200‐bp paired‐end sequencing following the Illumina protocol (Illumina version 3, San Diego, California, USA). Sequencing was performed by Novogene Biotechnology Company (Tianjin, China) on an Illumina HiSeq 2000 platform (Illumina), yielding 94,855,756 raw reads. All raw reads have been deposited into the National Center for Biotechnology Information's (NCBI) Sequence Read Archive (SRA; BioProject accession number: SRR8365238). Reads were filtered by trimming adapters and removing ambiguous reads (N > 10%) and low‐quality reads (>50% of nucleotides with *Q* value ≤ 5) using in‐house perl scripts, yielding 90,311,228 clean reads. Clean reads were assembled into 176,110 transcripts using Trinity v2012‐10‐05 (Grabherr et al., 2011) with the default parameters, and were then clustered into 63,763 unigenes using TGICL version 2.1 (Pertea et al., 2003).

MISA version 1.0 (Thiel et al., 2003) was used, with default settings, to detect SSRs. The criteria for identifying SSR motifs were as follows: the minimum number of nucleotide repeats was six for dinucleotide repeat motifs, and five for tri‐, tetra‐, penta‐, or hexanucleotide repeat motifs. In all, 9201 SSRs were retrieved, and 50 of them with five or more di‐, tri‐, tetra‐, penta‐, or hexanucleotide repeats were randomly selected for primer design using Primer3 software (Rozen and Skaletsky, 1999) according to the following parameters: (1) primer size from 20 to 25 bp and (2) product size from 100 to 280 bp. All primers were synthesized by Sangon Biotech (Shanghai, China).

We performed a preliminary screen of 50 EST‐SSR primers using three individuals each from four populations of *S. sinomontana* (Appendix 1). Total genomic DNA was extracted from silica‐dried leaves using the modified cetyltrimethylammonium bromide (CTAB) method of Doyle and Doyle (1987). PCR reactions were carried out in a total volume of 25 μL containing 2.0 μL of total genomic DNA (10–20 ng), 0.3 μL of each primer (10 pM), 0.3 μL (1.5 units) of *Taq* polymerase, 2.5 μL of 10× PCR buffer (with Mg^2+^), and 2.5 μL of 10 mM dNTPs. The PCR profile included an initial pretreatment of 10 min at 94°C; followed by 35 cycles of 1 min denaturation at 94°C, 50 s at locus‐specific annealing temperatures (Table 1), and 1 min elongation at 72°C; and a final extension at 72°C for 10 min. The PCR products were screened using 1% agarose electrophoresis to determine whether amplifications were successful for the expected sizes and then separated on 6% polyacrylamide gels. Overall, 19 of 50 (38%) EST‐SSR primer pairs produced clear, unique amplification products of the expected size. Of these, 13 loci were polymorphic across populations. Characteristics of all 19 loci are listed in Table 1. For all 13 polymorphic SSR loci, the 5′ end of each forward primer was labeled with one of three fluorescent dyes (FAM, HEX, or ROX; Table 1). PCR amplifications were then performed using 71 individuals from four populations of *S. sinomontana* with the same protocol described above (Table 2). The fluorescently tagged PCR products were analyzed on an ABI 3730xl DNA Analyzer (Applied Biosystems, Foster City, California, USA) with a GeneScan 500 LIZ Size Standard (Applied Biosystems), and allele sizes were scored with GeneMapper version 3.2 (Applied Biosystems). Number of alleles per locus (*A*), observed heterozygosity, and expected heterozygosity were calculated with POPGENE version 1.32 (Yeh et al., 1999). Hardy–Weinberg equilibrium was tested for each population using GENEPOP version 4.2 (Rousset, 2008). Using MICRO‐CHECKER version 2.2 (van Oosterhout et al., 2004), we found no evidence of null alleles across all loci.

**Table 1:** Characteristics of the 19 EST‐SSR markers developed for *Saxifraga sinomontana.*

Locus	Primer sequences (5′–3′)	Repeat motif	Allele size range (bp)	*T*_a_ (°C)	Fluorescent label	GenBank accession no.	BLASTX top hit description	*E*‐value	GenBank accession no. of BLASTX top hit
SS1	F: CGGAATCCTGTATGCGCTCT	(GA)_6_	244–298	53.5	FAM	MK348907	No significant similarity found	—	—
	R: AAACAAGTCCTCAACGACCA								
SS2	F: TCCACCCATAGGCCAAACAA	(AG)_8_	264–294	62	FAM	MK348908	No significant similarity found	—	—
	R: CACTACCACCGCCAACACTA								
SS8	F: TTTTGACTGGTGCTGGTGCT	(TCA)_6_	245–263	52	FAM	MK348910	Transcription factor TCP20 [*Cucumis sativus*]	5E‐12	XP_004148022.1
	R: GTGAGCGATTACTGCCCTGA								
SS9	F: CGCTTGTTCATAGGCTTCGC	(TTG)_6_	189–198	53.5	FAM	MK348911	Trihelix transcription factor ASIL1‐like [*Lupinus angustifolius*]	8E‐05	XP_019455555.1
	R: GCCTCCTGTTTCGGTCAAGA								
SS10	F: CCGCAGATCCGTTACCGAAA	(TTC)_5_	269–275	53.5	HEX	MK348912	No significant similarity found	—	—
	R: TGGTTCTGCAAAACGATGCT								
SS11	F: TGGATAGAGGCGAGGATCGT	(GTG)_8_	92–125	53.5	HEX	MK348913	No significant similarity found	—	—
	R: AGAAGTTCTGGGCGTTACGT								
SS16	F: ACGCAAAGGTAGGAGGAGTG	(AAT)_6_	244–286	53.5	HEX	MK348914	Cyclin P/U [*Corchorus capsularis*]	3E‐12	OMO60173.1
	R: AGTCCATTTCCTGAGGTGGTG								
SS32	F: TCCTACGTTTTGGAATCAAGGT	(GTTT)_5_	170–185	53.5	HEX	MK348920	No significant similarity found	—	—
	R: GCTCCGCCCCTGCTTAATTA								
SS35	F: GGGGAAAGAAATGGCTTCGC	(ATTG)_5_	250–339	56.5	ROX	MK348921	Hypothetical protein BCR41DRAFT_388307 [*Lobosporangium transversale*]	3.9	XP_021878789.1
	R: AGGGAGCTCCGAAAACACTT								
SS40	F: TCGGATTGGACTTGTTGGGG	(AAGGC)_5_	221–241	53.5	ROX	MK348922	No significant similarity found	—	—
	R: ATCGGGTCTAAGTCAGCCCT								
SS44	F: CCCTTCAGTTGGCGACTCAT	(CAAGA)_5_	109–130	53.5	ROX	MK348923	No significant similarity found	—	—
	R: TGTCTGCAACAATCCACAGGT								
SS46	F: ACAAATGCGGACACTGTGGA	(TTGCCC)_5_	328–402	56.5	ROX	MK348924	Phosphoglycerate mutase family protein [*Artemisia annua*]	2E‐16	PWA90260.1
	R: AGGCATTGATTCATTCAGGTGA								
SS47	F: CCATCTCGTGGCAGCAAAAC	(GACCAA)_9_	187–251	53.5	FAM	MK348925	No significant similarity found	—	—
	R: TTTGATTCGGGTTCAGGGGG								
SS6^a^	F: ACGCTTCATCATACAATGACTCA	(TA)_5_	233	53.5	—	MK348909	No significant similarity found	—	—
	R: CCAAGCAGTTCGGTGTCTCT								
SS24^a^	F: AGCTCCGTCCCAAGCTAGTA	(GGT)_6_	219	53.5	—	MK348915	Nuclear pore complex protein NUP88‐like isoform X1 [*Arachis hypogaea*]	6E‐04	XP_025622950.1
	R: TCCCCGACTTTCACTTCACG								
SS27^a^	F: ACGAGTGCTTTCGACATTTCC	(ATC)_6_	169	53.5	—	MK348916	No significant similarity found	—	—
	R: GGCGAAGATGCGTTTGATCC				—				
SS28^a^	F: ACATTTCCATCATCAACGCTTGT	(CACT)_5_	215	56.5	—	MK348917	No significant similarity found	—	—
	R: TGGAGATTGTAGGTAGTTGGTTGA								
SS29^a^	F: CGTGCTGCTGCTGATAAGGA	(TTGA)_5_	114	53.5	—	MK348918	60S ribosomal protein like [*Actinidia chinensis* var. *chinensis*]	0.001	PSS30694.1
	R: TCCACAAACCGAAATGATTGTCG								
SS31^a^	F: TGTGAGCTTGTAACTGCCAGT	(GACA)_6_	140	56.5	—	MK348919	DUF4153 domain‐containing protein [*Stenotrophomonas maltophilia*]	5.7	WP_100463508.1
	R: TCACATTCACGTGCTCTGCT								

Note

*T*_a_ = annealing temperature.

a Monomorphic loci.

**Table 2:** Genetic diversity of the 13 polymorphic loci across four *Saxifraga sinomontana* populations.^a^

Locus	Aba (*n* = 8)			Changdu (*n* = 28)			Chengduo (*n* = 18)			Dingri (*n* = 17)			Total (*n* = 71)		
	*A*	*H*_o_	*H*_e_	*A*	*H*_o_	*H*_e_	*A*	*H*_o_	*H*_e_	*A*	*H*_o_	*H*_e_	*A*	*H*_o_	*H*_e_
SS1	5	0.6250	0.6083	5	0.7500	0.7110	7	0.8333	0.6984*	2	1.0000	0.5152*	9	0.8169	0.7617
SS2	5	0.6250	0.7667	8	0.7500	0.8247	10	0.7222	0.8571	2	0.1176	0.1141	12	0.5775	0.8495
SS8	5	0.8750	0.7750*	6	0.7500	0.6117	5	0.8889	0.7444	2	1.0000	0.5152*	6	0.8592	0.7375
SS9	3	0.3750	0.4917	3	0.4643	0.4643	4	0.6111	0.5603	3	1.0000	0.6078*	4	0.6197	0.6610
SS10	2	0.1250	0.3250	2	0.3214	0.2747	3	0.7222	0.5222	1	0.0000	0.0000	3	0.3239	0.5063
SS11	7	0.6250	0.7417	5	0.6429	0.7188	4	0.5556	0.4968	3	0.1176	0.1159	7	0.4930	0.7472
SS16	3	0.5000	0.4250	2	0.2143	0.2494	5	0.7778	0.5841	3	1.0000	0.5473*	6	0.5775	0.4939
SS32	3	0.2500	0.5667*	4	0.4643	0.5591	4	0.6111	0.6270	2	1.0000	0.5152*	5	0.6056	0.7459
SS35	4	1.0000	0.6417	3	0.2500	0.2305	3	0.2778	0.2524	1	0.0000	0.0000	7	0.2817	0.2585
SS40	3	0.6250	0.5750	4	0.3571	0.4227	2	0.4444	0.3556	2	0.2353	0.2139	5	0.3803	0.3850
SS44	3	1.0000	0.5917*	3	0.7857	0.6370	3	1.0000	0.6413*	3	1.0000	0.6505*	4	0.9155	0.6933
SS46	13	0.8750	0.9750	5	0.7500	0.7221	8	0.8333	0.7413	2	0.0588	0.0588	18	0.6197	0.8305
SS47	7	0.7500	0.8417	8	0.8929	0.8357	6	0.7778	0.7365	2	1.0000	0.5152*	13	0.8732	0.8094
Mean	5	0.6346	0.6392	5	0.5687	0.5586	5	0.6966	0.5889	2	0.5792	0.0834	8	0.6111	0.6523

Note

*A* = total number of alleles per locus; *H*_e_ = expected heterozygosity; *H*_o_ = observed heterozygosity; *n* = number of individuals sampled.

a Population and voucher information are provided in Appendix 1.

* Significant departure from Hardy–Weinberg equilibrium at *P* < 0.05.

Among the 13 polymorphic loci, *A* ranged from three to 18 (mean = 8). The Dingri population had the lowest mean values (*A* = 2) among the four populations. Levels of observed and expected heterozygosity varied from 0.2817 to 0.9155 and 0.2585 to 0.8495, respectively, which indicates that genetic diversity is relatively high in this species. Additionally, a few loci showed significant deviations from Hardy–Weinberg equilibrium: three in the Aba population, two in the Chengduo population, and seven in the Dingri population (*P* < 0.05; Table 2).

All 13 EST‐SSR markers also amplified successfully in *S. tangutica*,* S. heleonastes*, and *S. congestiflora*, using the same PCR protocol as for *S. sinomontana* (Table 3).

**Table 3:** Genetic diversity in three congeneric species based on the 13 polymorphic microsatellite loci developed for *Saxifraga sinomontana*.^a^

Locus	*Saxifraga tangutica*						*Saxifraga heleonastes*						*Saxifraga congestiflora*					
	Cuona (*n* = 8)			Xinghai (*n* = 15)			Chengduo (*n* = 7)			Luozha (*n* = 4)			Dege (*n* = 7)			Shiqu (*n* = 6)		
	*A*	*H*_o_	*H*_e_	*A*	*H*_o_	*H*_e_	*A*	*H*_o_	*H*_e_	*A*	*H*_o_	*H*_e_	*A*	*H*_o_	*H*_e_	*A*	*H*_o_	*H*_e_
SS1	5	0.5000	0.6667	8	0.9333	0.7862	5	0.4286	0.5934	4	0.5000	0.8214	4	0.5714	0.6484	5	0.8000	0.7556
SS2	4	0.6250	0.6583	6	1.0000	0.8299	4	0.8571	0.6484	4	0.7500	0.7500	4	0.7143	0.5714	4	0.5000	0.6364
SS8	4	1.0000	0.6417	3	1.0000	0.5770*	2	1.0000	0.5385*	3	1.0000	0.7333	4	1.0000	0.6593	3	1.0000	0.6444
SS9	5	0.7500	0.6083	3	0.7333	0.6897	2	0.1429	0.1429	3	0.5000	0.7143	2	0.8571	0.5275	3	0.6667	0.6212
SS10	12	1.0000	0.9583	7	0.5333	0.5103	1	0.0000	0.2637	3	0.2500	0.4643	2	0.1429	0.4945	3	0.6667	0.5303
SS11	5	0.3750	0.6083*	4	1.0000	0.5973*	2	0.7143	0.4945	3	0.7500	0.6786	6	0.8571	0.8352	8	0.5000	0.9242*
SS16	2	0.1250	0.1250	2	0.9333	0.5149*	5	0.7143	0.8242	5	1.0000	0.8929	2	0.5714	0.4396	4	0.8000	0.7333
SS32	3	0.3750	0.3417	4	0.8667	0.6345	10	1.0000	0.9341	5	1.0000	0.9333	2	0.0000	0.2637	2	0.0000	0.3030
SS35	2	0.0000	0.2333	4	0.4000	0.3954	2	0.4286	0.3626	3	0.3333	0.6000	6	0.4286	0.8462*	4	0.2000	0.6444*
SS40	2	0.8750	0.5250	3	0.4000	0.4759	3	0.5714	0.5824	1	0.0000	0.0000	2	0.1429	0.3626	1	0.0000	0.3030
SS44	1	0.0000	0.0000	5	0.3333	0.4115*	7	0.8571	0.8901	4	1.0000	0.7857	3	1.0000	0.6044*	2	0.8333	0.5303
SS46	3	0.3333	0.6000	9	0.4167	0.8442*	2	0.0000	0.3556	5	1.0000	0.9333	6	0.1667	0.8636*	7	0.4000	0.9333*
SS47	7	1.0000	0.8833	10	1.0000	0.8529	3	0.7143	0.5824	5	0.7500	0.8929	6	1.0000	0.8352	7	1.0000	0.9643

Note

*A* = total number of alleles per locus; *H*_e_ = expected heterozygosity; *H*_o_ = observed heterozygosity; *n* = number of individuals sampled.

a Population and voucher information are provided in Appendix 1.

* Significant departure from Hardy–Weinberg equilibrium at *P* < 0.05.

## CONCLUSIONS

The 13 EST‐SSR markers developed here showed high polymorphism in *S. sinomontana* and high cross‐species amplification success. Hence, these are valuable loci for investigating genetic diversity, population structure, and evolutionary history in *S. sinomontana* and throughout *Saxifraga*.

## Data Availability

Raw sequencing reads were deposited in the National Center for Biotechnology Information (NCBI) Sequence Read Archive (BioProject SRR8365238). Sequence information for the developed primers has been deposited in NCBI's GenBank, and accession numbers are provided in Table 1.

## Appendix 1 Locality and voucher information for the populations of *Saxifraga* used in this study.

**Table:**

Species	Voucher no.	Collection locality	Geographic coordinates	Elevation (m)	*n*
*Saxifraga sinomontana* J. T. Pan & Gornall	Chen 2014558	Yushu, Qinghai	32°34′20.7″N, 97°12′41.6″E	4880	1
	Chen 2012317	Aba, Sichuan	32°46′02″N, 101°40′01″E	3450	8
	Chen 2014282	Changdu, Tibet	31°04′48″N, 96°56′59″E	4610	28
	Chen 2012347	Chengduo, Qinghai	33°12′02″N, 97°28′13″E	4450	18
	Chen 2007078	Dingri, Tibet	28°55′58″N, 87°26′24″E	5160	17
*Saxifraga tangutica* Engl.	Chen 2014409	Cuona, Tibet	28°19′23.4″N, 91°55′08.5″E	4770	8
	Chen 2007004	Xinghai, Qinghai	35°36′50″N, 99°32′05″E	3980	15
*Saxifraga heleonastes* Harry Sm.	Chen 2006024	Chengduo, Qinghai	34°07.457′N, 97°39.411′E	4850	7
	Chen 2014483	Luozha, Tibet	28°24′39.2″N, 90°34′31.4″E	5110	4
*Saxifraga congestiflora* Engl. & Irmsch.	Chen 2007226	Dege, Sichuan	31°57′25″N, 98°52′43″E	4180	7
	Chen 2007250	Shiqu, Sichuan	32°29′33″N, 98°27′17″E	4380	6

Note

*n* = number of individuals sampled.

Voucher specimens deposited at the herbarium of the Northwest Institute of Plateau Biology (HNWP), Xining, Qinghai, China.
